# Callers’ attitudes and experiences of UK breastfeeding helpline support

**DOI:** 10.1186/1746-4358-8-3

**Published:** 2013-04-29

**Authors:** Gill Thomson, Nicola Crossland

**Affiliations:** 1 Maternal & Infant Nutrition and Nurture Unit (MAINN), School of Health, University of Central Lancashire, Preston, PR1 2HE, UK

**Keywords:** Breastfeeding, Helpline, Peer support, Qualitative, Evaluation

## Abstract

**Background:**

Breastfeeding peer support, is considered to be a key intervention for increasing breastfeeding duration rates. Whilst a number of national organisations provide telephone based breastfeeding peer support, to date there have been no published evaluations into callers’ experiences and attitudes of this support. In this study we report on the descriptive and qualitative insights provided by 908 callers as part of an evaluation of UK-based breastfeeding helpline(s).

**Methods:**

A structured telephone interview, incorporating Likert scale responses and open-ended questions was undertaken with 908 callers over May to August, 2011 to explore callers’ experiences of the help and support received via the breastfeeding helpline(s).

**Results:**

Overall satisfaction with the helpline was high, with the vast majority of callers’ recalling positive experiences of the help and support received. Thematic analysis was undertaken on all qualitative and descriptive data recorded during the evaluation, contextualised within the main areas addressed within the interview schedule in terms of ‘contact with the helplines’; ‘experiences of the helpline service’, ‘perceived effectiveness of support provision’ and ‘impact on caller wellbeing’.

**Conclusion:**

Callers valued the opportunity for accessible, targeted, non-judgmental and convenient support. Whilst the telephone support did not necessarily influence women’s breastfeeding decisions, the support they received left them feeling reassured, confident and more determined to continue breastfeeding. We recommend extending the helpline service to ensure support can be accessed when needed, and ongoing training and support for volunteers. Further advertising and promotion of the service within wider demographic groups is warranted.

## Background

The postnatal period is a physical, emotional and social transitional period through which mothers typically need support to recover from childbirth and adjust to motherhood [1,2]. For many women, breastfeeding is a key area in which they require help and support. Currently the UK has one of the lowest breastfeeding rates in Europe, with only 1% of women exclusively breastfeeding their infants by six months of age [3]. Insights from a recent UK national survey suggest that up to 90% of women who stop breastfeeding in the early post-natal period wished they had breastfed for longer [3], and a lack of timely support for mothers has been identified as an important contributory factor for breastfeeding cessation [3,4]. In many societies, including the UK, as breastfeeding is not the cultural norm, and where geographical mobility tends to disperse families, women often have to look outside their own social networks for support to breastfeed their babies. This help may be provided by health professionals or by peer supporters (paid or voluntary).

Over the last decade, peer support has been the focus of a number of policy initiatives nationally and internationally to promote and support breastfeeding. Breastfeeding peer support has been identified as an important means to increase breastfeeding rates by the World Health Organization [5], the UK Department of Health (DH) [6] and the UK National Institute of Health and Clinical Excellence [7,8], and is supported by recent breastfeeding peer support commissioning guidelines [7]. Peer support can be described as “the provision of emotional, appraisal, and informational assistance by a created social network member who possesses experiential knowledge of a specific behaviour or stressor or similar characteristics as the target population” [9] p.329. Qualitative studies investigating women’s experiences of breastfeeding peer support in a variety of settings have found that women value receiving support from someone with shared experiences who can relate to their situation, who communicates in a familiar language and style, and who offers sufficient time [10-18].

A number of breastfeeding peer support programmes have included or specifically focussed on the impact and efficacy of telephone support. A systematic review of telephone support for women during pregnancy and the early postpartum period was undertaken by Dennis and Kingston. This review reported on three randomised controlled trials, conducted in Canada and the USA that evaluated the effect of proactive telephone support (peer or professional) on breastfeeding duration and exclusivity. Meta-analysis identified an overall positive effect on the continuation of both ‘any’ breastfeeding (three trials) and exclusive breastfeeding (two trials). In one trial, telephone support provided by health professionals did not lead to an increase in breastfeeding rates, while telephone support from peer supporters led to an increase in breastfeeding rates in two trials [19]. In a randomised controlled trial of telephone-based breastfeeding peer support for adolescent mothers, women in the intervention group received telephone support at two, four, and seven days, and two, three, four and five weeks after discharge from hospital. This study found that the duration of exclusive (rather than ‘any’) breastfeeding was increased in the intervention group relative to a control group receiving standard care [20]. However, an evaluation of a breastfeeding peer support programme in Hong Kong, in which women received one postnatal visit and then support was provided entirely through telephone contacts thereafter, found no significant differences in breastfeeding duration or exclusivity [21]. An Australian study of telephone-based breastfeeding support provided by health professionals (lactation consultants) found higher rates of exclusive breastfeeding at 4.5 weeks in the intervention group in one setting (a private hospital) with no differences in the other setting (a public hospital) and no differences in breastfeeding rates in either setting at three months, despite high levels of user satisfaction [22]. Taken together, these studies indicate that telephone support can increase breastfeeding rates, though outcomes are mixed and may differ depending on setting or provider.

Telephone support may also be user-initiated, such as in the form of a breastfeeding helpline. Recent evaluations of hospital breastfeeding helplines staffed by health professionals in the US [23] and Taiwan [24] described the characteristics of callers and consultations and reported high levels of usage. However, neither of these studies involved service-user evaluations of effectiveness or satisfaction with the support received. Janssen and colleagues’ conducted an evaluation of a Chinese-language infant feeding helpline in Canada. This study revealed high levels of need and suggested an increase in rates of exclusive breastfeeding following the introduction of the helpline service, but did not report any qualitative insights from service users [25]. To date the only study which has explored callers’ experiences of a breastfeeding helpline is an evaluation of the UK-based Drugs in Breastmilk Helpline undertaken by Rutter and Jones. This study involved interviews with 101 participants (77 service users and 24 health professionals) and the results revealed high levels of user satisfaction [26]. However, as this comprises a specialist service providing information on the use of medications while breastfeeding, these insights are not representative of callers’ insights towards more generalised breastfeeding help and support.

In the UK, there are four national organisations that provide breastfeeding helplines: the National Childbirth Trust (NCT), La Leche League (LLL), the Breastfeeding Network (BfN) and Association of Breastfeeding Mothers (ABM). These helplines are staffed by trained supporters, all of whom have breastfed their own children. Since February 2008, the UK Department of Health have funded a National Breastfeeding Helpline (NBH) which is provided by unpaid volunteers who are either a BfN Registered Breastfeeding Supporter, or an ABM Breastfeeding Counsellor. All Supporters/Counsellors who staff the helplines have completed an extensive period of training, and who receive ongoing training and support (please refer to our earlier publication for full details [27]). This service is available everyday across the UK from 9.30 am until 9.30 pm to provide breastfeeding information and support. As both of the breastfeeding organisations (BfN and ABM) wanted to retain their own helplines, as well as provide helpline services for the NBH, when helpline volunteers receive calls, these are from callers who had either contacted the helpline of their own organisation (BfN or ABM) or the NBH. Since the NBH was introduced, calls into the helpline have substantially increased (from approximately 9,000 in 2008 to over 35,000 during 2011), coupled with a dramatic reduction in calls to the BfN (from approximately 17,000 to 12,000) and ABM (from approximately 7,000 to 4,000) helplines.

In May 2011 our research unit was commissioned to undertake an evaluation of the NBH service. However, as the NBH helpline was integrated with the providers existing helpline services, the volunteer answering the call was unaware as to which helpline the caller has accessed, and callers received a ‘usual’ service, we decided to interview all callers into the helpline(s) who consented to take part, and to collect data to ascertain which helpline number they had called.

In a previous publication, we reported on a series of multiple regression models to elicit the variables associated with callers ‘overall satisfaction’ of the helpline service. Statistical modelling identified that the most important factors affecting satisfaction related to volunteers having sufficient time to deal with the callers’ issues; the information being perceived as helpful; the volunteers providing the support the callers needed; and for callers to feel reassured following the call [27].

In this paper, we present new insights into callers’ experiences of breastfeeding helpline support. Through a synthesis of the qualitative and descriptive data, thematic interpretations of callers’ experiences are reported to illuminate what and how the telephone support was internalised by callers, and the potential impact of this support on their breastfeeding experiences.

## Methods

Whilst detailed insights into the methodology used within the evaluation has been detailed within a former publication [27] an outline has been provided as follows.

### Design and interview development

A mixed-method design was used for this study within a structured telephone interview. Whilst the majority of questions posed during the interview were closed or utilised Likert scales (e.g. callers asked to respond to statements using a scale of 1 (very satisfied/strongly agree) to 5 (very dissatisfied/strongly disagree)), during the interviewer training it was agreed that any comments or feedback made by callers during their response to these questions would be recorded on the interview form. These statements explored callers’ experiences of accessing the helpline; their attitudes and perceived impact of the help and support received; and to what extent the support affected their wellbeing following the call. The interview also included a number of open questions to record any ‘benefits’ of contacting the helpline; what was ‘most’ and ‘least’ helpful as well as recommendations for service development.

Basic demographic information in relation to age, parity, ethnicity, marital status were also recorded, as well as infant feeding status and reason for the call.

The interview schedule was developed through a review of previous helpline literature, as well as research into breastfeeding peer support, discussions with the funders and volunteers who operate the helplines, and the Health Technologies Assessment guidance on the design and use of questionnaires [28]. The schedule was reviewed by six members of the University of Central Lancashire (methodologists (n = 2) and research staff (n = 4)) who have experience in survey development and/or research/evaluation into peer support and was trialled with four mothers. Revisions primarily related to question wording and sequence of questions rather than content changes.

Seven female interviewers undertook the telephone interviews. All interviewers were involved in a training event delivered by the project lead (GT). The first three weeks of data collection constituted a pilot phase to reflect on the recruitment strategy, sampling plan and interview schedule. Seventy-five interviews were undertaken over this period, following which minor alterations were made to the schedule to improve clarity and reduce repetition. The pilot data were included within the final data set.

### Ethics

Ethics approval was obtained from the BuSH (Built Environment, Sport and Health) Ethics Committee at the University of Central Lancashire (Proposal No.497) on 12^th^ May, 2011. Ethical issues in terms of informed consent, withdrawal and confidentiality were adhered to throughout this study.

### Recruitment

Over a 14-week period (May–August 2011) all the volunteers operating the helpline asked callers at the end of the call whether they would be willing to take part in a telephone evaluation. Whilst health professionals use the helpline(s), as the study aimed to elicit attitudes and effectiveness of the support and impact on caller outcomes, a decision was made to only recruit callers with direct experiences (e.g. mothers or a member of their personal network). Once consent had been provided, the volunteers recorded the date and time of call, the caller’s name, telephone number, which helpline they had contacted and name of the provider organisation (ABM or BfN).

### Data collection

On a weekly basis, regional BfN (n = 5) and ABM (n = 5) coordinators contacted all the volunteers in their area, and recorded all the details of the callers who had agreed to participate on a master data sheet, with all completed data sheets subsequently forward to the project lead (GT). Once received, any duplicate details were removed through checking the names/contact details with previous lists. It was also decided that any callers’ details received more than three weeks after the call had been made would be removed in order to limit recall bias. These occasions occurred when the coordinators experienced difficulties in contacting volunteers within the agreed weekly time-frame. The remaining caller details were then forwarded to the interviewers. Four attempts were made to contact each caller, at various times and days.

When contacted, the caller was provided with a standard script that provided information as to why the evaluation was being undertaken, and to establish consent for the interview. All the questions within the structured interview schedule were then read out, with answers hand-recorded on the interview form. Any qualitative responses or comments (e.g. whilst responding to the Likert based questions, or to the open-ended questions) made during the interview were recorded; with callers asked to wait to allow the interviewer sufficient time to record their answers, accompanied by clarification to ensure their views had been captured.

Any occasions where the participant was unable to be contacted (e.g. due to an incorrect telephone number being recorded) or the caller was not willing to participate was recorded. All completed, anonymised interview schedules were returned to the project lead.

### Data analysis

Analysis of quantitative data was performed using SPSS and Stata (v.11). All qualitative data including comments made during the interview and answers to open-ended questions were entered into a qualitative software package (MAXQDA). Thematic analysis was undertaken using the framework developed by Braun and Clarke [29]. This involved reading and re-reading of the qualitative data, noting and listing recurring concepts and sub-themes, mapping the data into meaning groups and networks and synthesis into key thematic areas. A further level of interpretation was also undertaken which involved integrating the descriptive responses to the Likert-based questions/statements within the thematic areas. Finally all the themes were mapped onto the key areas addressed during the interview schedule in terms of ‘contact with the helpline’, ‘experiences of the helpline service’, ‘perceived effectiveness of support provision’ and ‘impact on caller wellbeing’. Analysis was primarily undertaken by the lead author (GT), with cross-checking and ongoing discussions with NC until consensual validation had been obtained. At the end of the interview, all callers were asked whether they would like to receive a copy of the key findings. Overall, 372 (41%) requested and received a summary of the main findings (descriptive and thematic data), together with a request for feedback and/or further details if their views and experiences had not been represented. None of these callers provided any further information.

### Participants

During the evaluation period some 9,507 calls were made to the breastfeeding helpline(s), 3,529 of which were answered (37.1%). Overall, the details of 1,605 callers’ who agreed to take part in the evaluation were forwarded to the project lead (GT); 24 (1.5%) duplicate names and 99 (6.2%) names received more than three weeks after the helpline call were excluded. There were 78 (4.9%) incorrect telephone numbers, 50 (3.1%) callers declined to be interviewed, six (0.4%) callers were either out of the country or hospitalised, and one inappropriate call concerned a student calling about coursework (0.1%). Some 439 callers were not contacted due to either the caller not being available after four contact attempts or more than three weeks had elapsed since the index call was made. Overall, a total of 908 telephone interviews were conducted (13 of which were only partially completed due to the caller ending the call; with all data retained for analysis purposes).

## Results

Details of the caller demographics are included in Table 1. During the evaluation 885 telephone interviews were undertaken with mothers (97.5%), 17 with husbands/partners (1.9%), five with grandmothers (0.6%) and one with a sister (0.1%). Overall these data revealed that typical callers into the helpline are aged between 29 and 35 years, married/living together, are of a white ethnic background, are first-time mothers, with infants under one month of age. Calls that related to multiparous mothers indicated that the majority had breastfed a previous child. Furthermore, almost 90% of the mothers were providing their infants (either fully or partially) with breast milk at the time of the call. Of the 908 participants, 703 (77.4%) had called the NBH helpline, 116 (12.8%) had contacted the BfN service, 28 (3.1%) had called the ABM helpline and 61 (6.7%) could not remember which helpline they had contacted.

**Table 1:** Caller characteristics

**Caller characteristics**	**Frequency (%)**
**Age (years)**	
Under 20	3 (0.3)
20–24	47 (5.2)
25–29	200 (22.0)
30–34	352 (38.8)
35–39	236 (26.0)
40+	55 (6.1)
Missing	15 (1.7)
**Marital status**	
Married/living together	858 (94.5)
In relationship	17 (1.9)
Single/separated/divorced	19 (2.1)
Not recorded	14 (1.5)
**Ethnicity**	
White	778 (85.7)
Mixed	22 (2.4)
Asian/Asian British	63 (6.9)
Black/Black British	21 (2.3)
Chinese/Other	9 (1.0)
Not recorded	15 (1.7)
**Parity**	
First-time mothers	607 (66.9)
At least one previous child	288 (31.7)
Not recorded	13 (1.4)
**Age of child at time of call**	
Pregnant	5 (0.6)
Under 1 month	446 (49.1)
Between 1–5 months	340 (37.4)
Between 6–12 months	86 (9.5)
Over 12 months	16 (1.8)
Not recorded	15 (1.6)
**Current infant feeding practices**	
Exclusive/fully breastfeeding^1^	628 (69.2)
Mixed feeding (breast and artificial milk)	189 (20.8)
Formula feeding	68 (7.5)
Not applicable^2^	10 (1.1)
Missing	13 (1.4)
**Did mother breastfeed previous child/children (n = 301)**	
Breastfed previous child/children	241 (80.1)
Did not breastfeed previous child/children	44 (14.6)
Missing	16 (5.3)

^1^This includes 73 cases where the mother was breastfeeding and had introduced complementary foods.

^2^Relates to mothers whose children were over 12 months of age and who had stopped breastfeeding and mothers who were pregnant.

With regard to the reasons why the callers contacted the helpline, overall a total of 1,447 responses were recorded across the callers. Not surprising based on the demographics of the callers (i.e. first time parents), the most common reason for calling the helpline was difficulties with ‘positioning and attaching’ the baby at the breast (n = 364, 40.1%); followed by concerns about the mother’s milk supply 274 (30.2%) and difficulties relating to the infant’s behaviour 268 (29.5%).

Overall, the evaluation revealed that approximately 95% of callers were satisfied/very satisfied overall with the helpline service; callers reported high levels of satisfaction/agreement to the majority of Likert statements posed and 94.2% claimed that they would use the helpline again in the future. (Tables detailing all the Likert statements and participants’ responses are presented in the main published paper from this evaluation [27]). In the following section, the thematic interpretation of 863 (n = 95%) callers’ qualitative comments and descriptive responses to all the statements recorded during the evaluation are reported; together with a selection of key illuminating quotes. For each included quote, the initials of the interviewer are provided, together with the participant number. An overview of the key themes and sub-themes is detailed in Table 2.

**Table 2:** Overview of themes and sub-themes

**Theme**	**Sub-theme**
**Contact with the helpline**	Uncertainty of support
	Accessibility, immediacy and convenience
	Attitudes towards the opening hours
**Experiences of the helpline service**	Experiential knowledge
	One-to-one contact
	Anonymity and confidentiality
	Connected nature of relationships
	Time to support
**Perceived effectiveness of support provision**	Knowledge base of the volunteers and information provision
	Support on my terms
	Utility of support provided
	Need for face to face
	Making a difference
**Impact on caller wellbeing**	Eradicating doubts and enhancing wellbeing
	Reassurance
	Feeling empowered through options and knowledge

### Contact with the helpline

During the interview, participants were asked to indicate how many times they had to call the helpline before their call was answered, how easy or difficult it was to access this telephone support and their attitudes towards the helpline opening hours. In this section, the feedback provided by the callers is presented under the headings of ‘*uncertainty of support’, ‘accessibility, immediacy and convenience’* and *‘attitudes towards the opening hours’*.

#### Uncertainty of support

Whilst the majority of participants (75.4%; n = 685) reported that the call under consideration had been answered on the first attempt, callers’ experiences from all their previous (or even more recent) attempts were reflected upon during the interview. From qualitative responses provided by some 125 callers, a number of them reported ‘*mixed’* and *‘varied’* experiences in contacting the service, whereas others considered that delays in access the service were not problematic due to the nature of the call, or understandable due to the voluntary nature of the service. However, for others, the uncertainty of call response was considered problematic. Participants also provided negative feedback towards long periods waiting on hold and/or having to make numerous call attempts due to no response:

*‘It took a while for it to be answered, and was worried it wasn’t going to be’* (GT.56)

On some occasions this meant that callers were trying over a number of days to access support – ‘*it took three days to speak to someone and got hung up on once’* (NC.95); or even that when the call was eventually answered, it was inconvenient for the caller to talk.

Delays in gaining access were cited as a key negative feature of the helpline service. Participants stressed that a helpline is used when callers are *‘desperate’* for help and support; it was therefore *‘frustrating’*, ‘*unhelpful’,* ‘*stressful’* and ‘*not reassuring’* when their calls went unanswered:

*‘It’s frustrating as you can’t leave a message, it took from 9.30 am to 1 pm and not knowing if you would get through’* (HC.199)

Furthermore, for several of the callers, this was cited as the main reason why they would not use this service in the future:

*‘Wouldn't use again due to difficulties in getting through, causes too much stress, it’s very hard to get through’* (NC.107)

#### Accessibility, immediacy and convenience

Overall, 85.5% (n = 776) callers strongly agreed/agreed that the opening hours enable people to access support when required. Additionally, over 200 callers referred to the merits of being able to access a service when they need it, acquiring immediate help and support from within the convenience of their own home. Callers often utilise the helpline when they are at their ‘*wits end’* and they ‘*really need to talk to someone’*. Participants repeatedly emphasised the limitations of locally available support networks due to the timing, location and/or availability of these services:

*‘There are groups and breastfeeding counsellors but only at set times and working hours and you need someone there in the early days of feeding’* (GT.110).

*‘Really good to have the helpline in areas where they haven't got much support groups. You can get advice when you need it’* (GT.105)

Callers also highlighted difficulties in leaving the home environment with a new baby, particularly for those who had had an operative birth or had older children:

*‘There and then being able to speak to someone, all going through my head so good to have someone there, no hassle, or babysitter or having to get out of the house to speak to someone’* (GT.54)

The helpline thereby offered a service which women could access during anti-social periods and without the complications and limitations involved in accessing other breastfeeding support networks (e.g. health visitors, midwives, GPs, breastfeeding clinics):

*‘Really hard to get hold of the midwife, you leave messages and they don’t call you back. If need immediate answers you have someone there from the comfort of your own home’* (GT.159)

Some of the callers also considered the efficiency (resource and cost-based) of helpline support, particularly for more *‘straight forward’* or *‘quick questions’*; as this meant that they did not have to seek out health professional guidance:

*‘Being able to call someone when worried rather than calling hospital especially since I didn't feel it was important enough to call the doctor or hospital’* (NC.102)

#### Attitudes towards the opening hours

A total of 800 (88.1%) callers considered it ‘very easy’ or ‘easy’ to make contact with the service. However, overall a third of the participants (37.4%; n = 340) believed that the opening hours of the helpline should be extended. The qualitative remarks presented varying perspectives towards this aspect of this service. A number of the participants (n = 67) considered the hours to be *‘fine as it is’.* As the helpline covers the early evening period, and is available over the weekend and holiday periods, the opening hours were deemed to be more than sufficient to meet families’ needs:

*‘The cover is enough for volunteers to be there when needed. Issues can be dealt with during the day’* (GT.01)

An inherent suggestion across these accounts was that people can wait until the helpline is open or should utilise other support options available to them (e.g. maternity unit, accident & emergency) if ‘*desperate’*.

Other participants considered that it was ‘*unrealistic to expect more’* due to the voluntary nature of the helpline service. A substantial number of participants were hesitant to suggest longer opening hours (irrespective of whether they considered this was needed) because they did not perceive it feasible or realistic to ask volunteers to provide an extended service:

*‘The ideal would be 24 hours but appreciate that volunteers would have to be on call at night, so I am happy with the service’* (FD.06)

However, from a counter perspective, and more commonly identified, were explicit statements emphasising the need to extend the opening times/periods of the helpline service, with 350 responses requesting earlier and/or later opening hours or even for a 24 hour service to be offered. Therefore, whilst the availability of the helpline outside of working hours was positively perceived, these additional comments accentuated the periods when there was ‘*absolutely no one’* there to help them: when callers were unable to contact friends, family or health professionals and no other dedicated breastfeeding support networks were available. These participants repeatedly highlighted that breastfeeding mothers experience more difficulties late evening and/or the middle of the night when they are ‘*pacing the house’*, ‘*in despair’* and ‘*feeling alone’* during a ‘*dark time’*:

*‘I tried after 9.30 pm as I needed to call at night, there was no help when I was so desperate’* (FD.01)

Moreover, a small number reflected that had this extended service been available, their difficulties may not have escalated:

*‘I needed help at 2 am as my breasts were engorged, if I had had help then I wouldn’t have developed mastitis – we need 24 hour help’* (GT.137)

The suggestions for extending the opening hours as well as the issues raised previously about calls being unanswered centred on callers being able to access support ‘*when they need it’*.

### Experiences of the helpline service

During the interview, participants were asked a series of statements to ascertain their experiences of the help and support received. The responses to these statements and key issues that emerged from the qualitative responses are discussed under the headings of *‘experiential knowledge’, ‘one-to-one contact, ‘anonymity and confidentiality’, ‘connected nature of relationships’* and *‘time to support’.*

#### Experiential knowledge

During the interview callers were asked to identify whether they knew that all the volunteers operating the helpline were breastfeeding/had breastfed their own child(ren). If the caller answered in the affirmative, they were subsequently asked whether they liked the opportunity of being able to speak to a breastfeeding mother. The data revealed that just over half the sample (54.7%; n = 497) were aware that the volunteers were/had been breastfeeding mothers, 96.8% (n = 481) of whom liked having the opportunity to discuss breastfeeding with someone who had breastfed themselves. Furthermore, 95.5% (n = 867) and 92.3% (n = 838) of callers strongly agreed/agreed that the volunteer understood what they were talking about and how they were feeling.

The limited number of occasions where callers were uncertain or did not agree with these statements (approximately 5% of occasions) generally related to callers wanting to speak to someone and therefore *‘not necessary that I speak to a breastfeeding mother’;* or the fact that the volunteers only appeared to offer their own experience, rather than from a wider theoretical knowledge base:

*‘She just talked about her personal experience rather than from a knowledge base’* (HC.34)

In the majority of occasions, however, callers were highly positive about discussing their experiences and issues with another mother, who *‘had done it themselves’*. Recurrent issues from over 160 participants related to callers being able to share their personal experiences with someone who had had a ‘*similar experience’* to them. Whilst the theoretical knowledge base of volunteers was important, the volunteers’ experiential knowledge meant they could empathise with the woman’s specific situation. The ‘*mother to mother’* support enabled a deeper appreciation and comprehension of what callers were ‘*going through’* and how they ‘*were feeling’*. These features were repeatedly considered to be the most beneficial attributes of the helpline service:

*‘Speaking to someone who has breastfed is great, who knows the emotions behind what breastfeeding is, the problems with breastfeeding and not just relaying information’* (PM.51)

*‘All such wise women who know where you are coming from’* (GT.36)

This shared knowledge base meant that volunteers were able to *‘talk on my level’,* as well as provide ‘*personal recommendations’*; with a number of callers highlighting the difficulties in discussing, or seeking support from family/friends or other professionals who did not have these shared experiences, knowledge or values towards breastfeeding:

*‘They show you in hospital, the basics and then you are sent home on your own and you don’t necessarily have breastfeeding people around you to help, so to be able to talk to someone and keep you going when you are exhausted and at crisis point, and particularly when people* (mother-in-law) *are trying to get you to give baby a bottle, it’s good to talk to someone who is encouraging breastfeeding’* (GT.160)

### One-to-one contact

Overall some 89.9% (n = 816) of callers strongly agreed/agreed that they found it easy to talk about their breastfeeding issues over the telephone. Aside from the immediacy and convenience of the support, over 110 participants frequently recited the value of being able to talk to someone about their concerns and issues. The opportunities for *‘one to one’,* ‘*human contact’* to ‘*get things off my chest’,* to ‘*talk things through’,* to be *‘listened to’,* and to *‘have my questions answered’* offered emotional as well as practical benefits for callers:

*‘Great to bounce around your own personal situation’* (GT.15)

*‘Just talking to someone always helps and makes me feel lighter and more refreshed. Gets me further than where I was’* (GT.39)

The value of being able to pick up the phone and talk to someone was also emphasised amongst callers with limited social networks, and amid those who had more unusual breastfeeding experiences (e.g. longer-term breastfeeding):

*‘It was the only place I could get information about breastfeeding a baby at 11 months, everywhere gears information around the first few weeks’* (KB.95)

#### Anonymity and confidentiality

Callers were requested to report whether they liked talking to a volunteer who did not know them and 56.7% (n = 515) of the participants either disagreed or provided an uncertain response to this statement. These callers often reported that ‘*they didn’t mind’* who they spoke to so long as suitable support was provided. Other callers praised the anonymous and confidential nature of the service (42.6%, n = 387). With qualitative feedback provided by some 34 callers identifying how they appreciated the *‘professional’* perspective from someone who had no presuppositions or opinions concerning their situation, and also the knowledge that their particular issue or situation would not be documented in ‘official’ records:

*‘I could call the midwife but I didn't feel comfortable as I did with the volunteer…. rather than someone in authority who can judge me, it’s a stranger so you haven't got the fear of everyone knowing my issues’* (GT.146)

*‘Can call triage if need to but I didn't want to call the hospital as they tend to log your calls which put me off as we had feeding issues earlier on, so I became suspicious of records’* (GT.49)

Some of the participants specifically highlighted how the remote nature of this service enabled them to ‘*talk more freely’* and to ask what they construed as *‘silly’* or ‘*embarrassing’* questions without fear of reprisals:

*‘You worry if you are being silly but it’s good as the person doesn't know you so you can off load’* (NC.29)

### Connected nature of relationships

Various statements were presented to elicit insights into the nature of the helpline service. From the qualitative responses, in only a few occasions (n = 8) were more negative terms such as *‘uncaring’*, *‘not helpful’* and *‘unapproachable’* used to describe the volunteer(s). However, in the additional feedback provided by over 110 callers, participants frequently used a range of positively charged characteristics, such as *‘ ‘patient’, ‘realistic’, ‘kind’, ‘wonderful’, ‘helpful’ ‘compassionate’, ‘empathic’ and ‘encouraging’* and a ‘*life saver’* to describe the volunteer(s).

Almost all (98.6%; n = 895) callers considered the volunteer to have treated them with respect, and 98.5% (n = 895) felt comfortable talking to the volunteer about their breastfeeding questions and/or concerns. Furthermore, from the positive qualitative feedback collated, participants frequently characterised a connected relationship with the volunteer, with many symbolising their interactions as a ‘*friendship’*.

*‘The volunteer was friendly, kind and helpful and informal. I felt like I was talking to a friend’* (KB.202)

Participants referred to the holistic person-centred approach of this service witnessed through the concern and interest expressed towards them and their families:

*‘I felt she really wanted to help me’* (PM.28)

Some of these participants emphasised how *‘nervous’* and *‘apprehensive’* they had been calling the helpline service. However, the volunteers approach was identified to have made the callers feel ‘*comfortable’* and ‘*at ease’*

*‘Talking to a mum in their home, I was a bit dubious about it at first, worried about bothering them but she was lovely and answered all my questions’* (GT.05)

### Time to support

Almost all of the participants (97.4%; n = 885) considered that the volunteer had enough time for them during the call. Those who expressed uncertainty or dissatisfaction (2%; n = 18) towards this issue reported feeling *‘rushed’* off the phone. However, far more commonplace were positive reactions, provided by over 70 callers in relation to how the volunteer had ‘*had time for them’* to *‘listen’* to their concerns and to provide them with the necessary support:

*‘I was really taken back by her concern and interest, it had no limits, she was happy to stay on the phone as long as I needed’* (GT.65)

Some participants reflected on how volunteers gave them ‘*all the time in the world’* and how they ‘*could not give me enough’*; providing space and time for their issues or concerns to unfold:

*‘I was on the phone for 1.5 hours, she could not have been more helpful’* (KB.143)

### Perceived effectiveness of support provision

Additional statements to ascertain the actual impact and influences of the support on their infant feeding experiences were presented during the interview. The key themes identified relate to *‘knowledge base of volunteers and information provision’, support on my terms’, ‘utility of support provided’, ‘need for face-to-face’ and ‘making a difference’.*

### Knowledge base of volunteers and information provision

Overall 92.3% (n = 838) of the callers strongly agreed/agreed that their questions had been answered and 94.1% (n = 855) reported that the information they had received had been helpful. Callers also offered positive remarks towards the knowledge-base of the volunteers, with 87.7% (n = 796) considering the volunteer to be an ‘expert’ in breastfeeding issues. From over 320 comments provided by the callers, participants depicted the helpline as a service through which they were able to receive *‘confident’*, *‘reliable’, ‘research-based’* and ‘*professional advice’*; which they could ‘*trust’* and have ‘*confidence’* in:

*‘Someone on the end of the phone who knows what you are talking about really spurs you on – having an expert on tap’* (GT.150)

Positivity was associated with direct answers to questions as well as ‘*clear’* and *‘useful’* tips, strategies and solutions, with numerous callers making reference to the practical as well as emotional based support they received:

*‘The person was very knowledgeable and information I received was excellent and research based. She gave me a lot of information and not rushed, very impressed’* (HC.60)

A central issue to emerge related to the way in which the volunteers tailored and transmitted information. Callers highlighted how the volunteer had asked questions to explore their history and previous practices (from the mother’s and infant’s perspective) which subsequently enabled important insights into their current feeding practices to be detected and appreciated:

*‘She asked me questions about the birth, and she asked me a lot of questions to find out what the issues were and made me more aware of the issues – If I hadn't have called the helpline I would probably be still pulling my hair out at home and wouldn’t have got to the bottom of the issues’* (GT.146)

Indeed, several participants recounted how the volunteer had, based on their verbal descriptions, identified issues and offered a range of practical and comprehensible solutions:

*‘Good to describe problems and get tips and said it might be tongue tie which turned out to be true’* (GT.27)

Participants who expressed uncertainty or disagreed to the statements concerning the expert status of volunteers (11.5%; n = 105), their questions being answered (7.2%; n = 65) and/or helpfulness of the information provided (5.2%; n = 47) occurred when volunteers were unable to provide support to resolve the women’s specific concerns. From the more negative feedback reported by over 100 participants, as callers were unaware of the background of the volunteers, or training they had received, this could lead to ambiguity in what information and help could be offered, with various participants left wishing that they had been able to access a more recognised specialist service:

*‘Information was too vague, and different to what I had been told, and I didn’t know the volunteer’s credentials whereas people at the clinic are specialist so it is hard to know who to trust, probably the clinic is more reliable’* (NC.23)

Dissatisfaction generally concerned the volunteer’s inability to answer their questions; the basic level of information provided; the information conflicting with guidance previously acquired and/or already being aware of the suggestions or advice proffered:

*‘I found it difficult over the phone and was directed to a website. I wanted something more definitive, it was too vague, too many different bits of information and differing advice’* (NC.23)

*‘I didn’t feel very reassured that the volunteer knew what the answer was. I couldn’t get an answer to the problem and I had already tried what was suggested’* (HC.28)

Several callers also reported on the impractical or inappropriate suggestions and support they had received; with volunteers occasionally discussing issues and offering counsels irrespective of the caller’s values, needs and beliefs:

*‘The advice was not practical, you have got to realise that not everyone is mother earth, I was advised to sit for as long as necessary with baby skin-to-skin which I could not do with a three year old and a funeral to organise’* (HC.237)

Other more critical comments concerned volunteers jumping to conclusions due to not listening or asking *‘the right’* questions, with several callers feeling bombarded by the extent of material relayed. These participants complained about being unable to remember and retain all the points discussed during the phone call, or feeling that the amount of information provided was unnecessary or even inappropriate for their specific situation:

‘*I had a specific problem and was told to do re birthing techniques and how to latch which I knew and did not need to go back over, totally inappropriate advice’* (HC.252)

As mothers often call in a highly emotional state, their capacity to retain detailed information becomes restricted. A few complaints were raised about the volunteers not clarifying the caller’s level of comprehension as reflected by the following mother:

*‘I felt the support could have been better, she gave me technical advice on how to express and latch onto an engorged breast, I thought I had to massage when feeding, I didn’t appreciate I needed to massage at other times and I found I was just waiting for the next feed. The next day I developed mastitis and had to get medication. I was panicking and in an emotional state and she assumed I was doing things that I didn't as I had missed that communication’* (GT.137)

#### Support on my terms

Some 93.0% (n = 844) of the callers strongly agreed/agreed that the helpline service had provided them with the support they needed, with just over 90% (n = 825) of callers considering the support to have met (or even ‘*exceeded’*) their expectations. Qualitative feedback from almost 80 participants divulged how the volunteer had worked with them to evaluate their own personal situation; to empower and support them to make choices that were best for themselves, their baby and their family, often irrespective of infant feeding method:

*‘She helped me evaluate my situation and allowed me to make choices of how to move forward to decide what was best for my baby and myself’* (HC.248)

Overall, 85.5% (n = 776) of the participants felt that the volunteer had encouraged them to continue breastfeeding, and 93.1% (n = 846) considered that the volunteer had made them feel it was OK to continue breastfeeding. Ambivalent responses to these questions (3.2%; n = 29 and 0.7%; n = 6 respectively) often reflected the nature of the call (e.g. breastfeeding discontinuation) rather than a lack of encouragement per se. Nonetheless, even though callers received and internalised this encouragement, a repeated message across many of the participants was how they did not ‘*feel pressurised’* to continue:

*‘Didn’t make me feel I had to continue, she was encouraging but not pushy’* (HC.90)

Many participants called the helpline for information and/or help in introducing artificial milks, using bottles (e.g. expressed breast milk), nipple shields or the cessation of breastfeeding. From the feedback provided, volunteers were identified to discuss and consider alternatives with women and to provide support and reassurance for their choices:

*‘Said it was OK to give a bottle and gave me praise for still continuing to breastfeed’* (GT.97)

Approximately 40 callers specifically made reference to the ‘*impartial’,* ‘*unbiased*’ ‘*non-judgemental’* support that prevented and protected against maternal guilt. Participants were pleasantly surprised to not have breastfeeding ‘*rammed down their throats’* nor encountering a solely ‘*earth mother’* approach. Moreover, whilst this response had not necessarily been expected, it was gratefully received:

*‘She didn't ram it* (breastfeeding) *down my throat. She was calm, balanced, perfect really, encouraged me and made me feel if I didn’t (*breastfeed*) that was still OK. It just what I needed’* (HC.66)

In contrast, other callers, (albeit a much smaller number, n = 11) claimed to have experienced more prejudiced views and attitudes when alternatives to exclusive or partial breastfeeding were mentioned. A few participants reported receiving ‘*disapproving’* and *‘judgemental’* attitudes and support:

*‘I felt she was quite disapproving of my decision to wean off breast milk onto formula. I wanted to mix feed and she was judgemental by her tone of voice and kind of “why wasn't I trying harder”'* (GT.106)

A small number of these participants referred to the *‘militant’* and *‘dogmatic’* attitudes expressed by the volunteers. This one-sided approach to breastfeed *‘at all costs’* was considered unhelpful and had the potential to induce self-reproach:

*‘A bit militant, combination feeding has been much better for me, she made me feel it was not OK for me to stop. She did not understand the issue and would not accept the baby had reflux. She said it was a very trendy word these days. I thought she was very single minded, very hard line and did not consider my requirements’* (HC.226)

Further issues to emerge related to callers not receiving the type of support that they specifically needed (n = 25). For example, there were instances of callers being provided with practical rather than the desired emotional support, or purely emotional support when more pragmatic based guidance was required:

‘*Felt it was more person-centred and I wanted instructional advice. There was a lot of time before I was getting anything out of it. I wanted ideas and suggestions’* (GT.35)

*‘I was not praised or encouraged and would have been nice to have that’* (HC.57)

#### Utility of support provided

Overall 85.7% (n = 778) of callers agreed/strongly agreed with the statement that they had been able to put into practice the information provided, with 75% (n = 681) considering that the support had helped them resolve their issues. Feedback from over 70 callers referred to how *‘helpful’* and *‘useful’* the solutions and suggestions had been, with numerous callers citing specific remedial action and the benefits of such:

*‘The volunteer talked through the information step by step, the positions and what to expect. I felt an improvement in terms of a loss of pain and baby feeding better and quicker by the next feed’* (KB.206)

For a number of the callers, however, despite expressing satisfaction towards the support, their issues and/or difficulties were still partially or fully ongoing. With 199 responses reflecting how callers had either not yet, or been able to implement the guidance provided:

*‘I tried to follow the advice but it didn’t work for her. I still felt I wasn't producing enough. I have had a lot of input but none of it was as helpful as the volunteer on the helpline’* (KB.193)

With some discontinuing breastfeeding due to their issues escalating into more serious clinical complications:

*‘Issues are still ongoing, the advice helped a bit as she suggested skin-to-skin, having a bath, trying to relax, various options but then the issues have continued’* (NC.34)

Those who were more ambivalent or negative about being able to put into practice the practical help received (8.9%; n = 81) or the extent to which it was able to resolve their particular issues (21.9%; n = 199) often reiterated points previously discussed in relation to the support not being *‘helpful’* or ‘*effective’*, with some participants indicating a lack of confidence or belief in the support provided:

*‘The volunteer had an answer for each question and reassured that its normal but she couldn’t explain why the baby isn’t settled, and she also discouraged me from topping up with formula I still feel the baby is not getting enough milk so I am topping up with formula’* (NC.148)

Several participants also reported that they had to seek out further clinical based support for help with their issues.

### Need for face-to-face

Whilst callers found it easy to discuss their breastfeeding issues over the telephone (89.9%; n = 816), one of the key limitations expressed by 123 callers concerned the remote nature of telephone support. The fact that volunteers were not able to observe the woman and her baby feeding is an obvious limitation to a very practice-based activity. Whilst this does not detract from the highly positive feedback received towards this service, these callers expressed difficulties in trying to implement what the volunteer was describing and how they needed ‘*someone there’* to ‘*show me what to do’*:

*‘She was brilliant but I don’t think it is the answer to the problem. From my experience and experience of friends you need physical support. The help and advice on things I did not know about and practical help and tips were great, but unfortunately it did not work’* (HC.193)

With numerous callers identifying how the problem was only resolved when subsequent face-to-face based support was accessed.

#### Making a difference

From the demographic data recorded, 90.0% of the women were continuing to provide their infant with ‘any’ breast milk at the time of the evaluation. However, two-thirds of the callers (66.8%; n = 606) were ambivalent or disagreed with the statement that they would have not have been able to carry on breastfeeding if they had not called the helpline. This was primarily related to calls concerning breastfeeding cessation, or questions unrelated to breastfeeding continuation (such as expressing and storage of breast milk, or managing night-time feeds). Over 140 callers’ reported that they ‘*would have* (breastfed) *anyway’*, with many of them citing that should their issue not have resolved, other support mechanisms would have been (and in a number of occasions were) sought:

*‘I would have waited to speak to someone. It wouldn’t have stopped me breastfeeding’* (GT.11)

Overall, however, just over 20% (n = 188) of callers felt that the support received via the helpline (practical and/or emotional) played a critical role in their breastfeeding continuation. With qualitative feedback from 134 participants identifying the difference that the support made to the breastfeeding experiences:

*‘The helpline was the last port of call for help. If it wasn't for that phone call I would have definitely given up breastfeeding’* (KB.206)

These participants recounted how they were on the verge of *‘giving up’* or ‘*reaching for the formula’* when they made the call. However, after they had talked the issues through and been provided with solutions and reassurance, these women continued to exclusively breastfeed:

*‘She was brilliant, amazing, a life saver, I was literally about to open a carton of formula and she saved my baby from having formula. This was down to her being calm, reassuring, talking to me and telling me that my milk hadn't dried up and my body was regulating the amount of milk’* (GT.128)

It is also important to consider that whilst the majority of callers were more ambivalent about the impact of the helpline support on their decision to breastfeed, almost 85% (n = 771) of the callers considered that the volunteer and/or support they received had encouraged them to continue breastfeeding, and 69.5% (n = 631) were more determined to continue breastfeeding after the call had been made. The support they received often helped them to resolve their issue(s) and make breastfeeding *‘easier’*, as well as providing encouragement and reassurance instilling confidence in women to ‘*keep going’* and/or persist until other support options could be accessed:

*‘Medical issues have emerged since but she put me in the right frame of mind to seek out further advice and help. When I called I felt I couldn’t put her to the breast as it was too painful but the support and different techniques helped me to continue’* (GT.146)

### Impact on caller wellbeing

The statements and responses concerning caller’s emotional and cognitive based responses following the call have been discussed and described under the sub-themes of *‘eradicating doubts and enhancing wellbeing’*, *‘reassurance’* and *‘empowering callers through knowledge and options’*.

### Eradicating doubts and enhancing wellbeing

Overall the findings revealed how the helpline made a significant impact on caller wellbeing, with callers feeling less worried (88.2%; n = 801), less stressed (86.2%; n = 783) and more confident (85%; n = 772) following the call. Positive feedback from 115 callers related to how the support had enabled them to *‘resolve their anxiety’*, to *‘relax’*, *‘de-stress’*, feel *‘calm’*, *‘confident’* and feel more *‘in control’* over their situation. The positive interactions with the volunteer promoting wellbeing in terms of self-esteem and self-beliefs:

*‘Really good. She gave me time and made me feel at ease, good tips and hints and made me feel I could carry on and made me feel really good’* (HC.182)

As previously identified, callers frequently called at points of high anxiety and distress and were grateful for the praise, encouragement and practical-based support received. Callers spoke of how the helpline eradicated their self-doubts, and provided them with *‘hope’* and a *‘boost’* leaving them feeling *‘happier’ ‘energised’* and even a *‘better mother’*.

Whilst there were only a few occasions in which participants provided qualitative comments to justify why they felt less certain or even disagreed with these statements (n = 15); these comments tended to concern the impractical nature of the support and/or judgemental support women received leading to feelings of *‘guilt’* and marginalisation. Moreover, in some cases, callers disagreed with these statements because the volunteer had identified an underlying medical condition requiring further treatment, thus exacerbating their emotional distress:

*‘It raised more issues and that made me more worried. The information she gave was good advice and I understood what and why she said it but it raised issues that I needed to consider and discussed with my health visitor’* (HC.210)

### Reassurance

Almost all of the participants (92%; n = 835) claimed that they felt reassured following the call to the helpline. Furthermore over 400 callers provided qualitative feedback that concerned why and how reassurance was experienced. From these comments, reassurance was specifically associated with issues such as being able to ‘*talk through my issues*’ with someone who knew and understood breastfeeding, and receiving *‘expert’* or *‘professional’* support for their specific issues:

*‘Getting the specific information that reassured me and finding out the information and calling me back to give me detailed information. Even though the GP reassured me it was the very specific and detailed information about the drug action in the amount of breast milk that the volunteer obtained that really reassured me’* (HC.106)

Callers also frequently highlighted how the helpline operated as a *‘lifeline’* through knowing that they could talk to someone who would appreciate and understand how they felt and who would offer support and guidance. As a consequence, this led to them feeling less *‘depressed’*, *‘lost’* and ‘*alone’*:

*‘Just knowing in the future there is someone to speak to, it’s very reassuring. You can read so much conflicting information on web sites and to be able to pick up a phone and speak to someone is fantastic’* (HC.08)

Another aspect of reassurance was through knowing that there was ‘*nothing wrong’* with their infants and the affirmation that they were doing *‘everything right’*; through the acceptance and validation of women’s choices and decisions and the normalisation experienced through the recognition that other women had similar feelings and had faced similar experiences:

*‘She made me feel that I was not the only person in the world having this problem, I failed to feed my daughter and I really wanted to breastfeed and when I had problems and thought breastfeeding was natural, I thought it must be me. So having someone explain it is normal and not completely easy for everyone’* (GT.126)

### Feeling empowered through options and knowledge

Some 77.2% (n = 701) of the participants considered themselves to be ‘more knowledgeable’ about breastfeeding after their call to the helpline. Those who offered more hesitant or negative feedback related to the caller already feeling knowledgeable or that the service did not provide them with ‘*anything I didn’t already know’*. However, over 70 of the callers who strongly agreed/agreed with this statement referred to how the information had enabled them to feel more confident through being able to understand and appreciate the underlying reasons for their specific issue (as well as preparing them for future occurrences, e.g. mastitis):

*‘She explained to me why my baby was breastfeeding frequently, this helped me to understand and become more patient with my baby’* (KB.182)

The information provided was often depicted as confirming or prompting what the callers already knew as well as providing them with new knowledge:

*‘Wealth of knowledge and feel that they are able to drawn on a much wider range of expertise and come up with ideas I had not thought of before’* (GT.22)

The information imparted was explicitly viewed as empowering, as some callers expressed how they were or would go on to use this knowledge to challenge other health professionals as well as familial-based advice:

*‘Felt more confident in knowledge they provided and to be able to go back to the GP with link to website re medication for baby as well’* (GT.157)

## Discussion

In this paper we have reported on the qualitative insights from an evaluation of UK breastfeeding helplines. Key thematic areas were identified and mapped against the main areas explored during the evaluation in terms of callers’ attitudes and experiences towards ‘contact with the helplines’, ‘experiences of the helpline service’, ‘perceived effectiveness of support provision’ and ‘impact on caller wellbeing’. Overall satisfaction with the helpline was high with the majority of callers recalling positive experiences. These findings indicate how callers valued the opportunity for accessible and convenient support to be provided at a time that they needed it; to be able to talk to someone who was impartial, evidence based and professional; and for information to be targeted to the needs of themselves and their families. In the majority of occasions, callers were able to put into practice the information and support provided to positive effect. Furthermore, whilst the insights suggest that telephone breastfeeding support alone does not necessarily influence a woman’s decision to continue breastfeeding, access to information, support and encouragement left the majority of callers feeling reassured, more knowledgeable, confident, motivated and more determined to breastfeed their infants.

This is the first evaluation of general, helpline-based breastfeeding support to report qualitative data on callers’ experiences of using the service. A large number of participants were recruited, thereby increasing the generalisability of the findings and due to the combination of descriptive and qualitative data, in-depth insights into positive and negative features of this support method have been revealed. All interviewers took part in a training event, and data input was undertaken by the project lead on an ongoing basis to ensure consistency, and minimize bias in how the information was recorded.

Our findings resonate with those from other qualitative studies exploring women’s experiences of breastfeeding support. During the evaluation callers used terms such as ‘understanding’, ‘warm’, and ‘kind’, or conversely, ‘uncaring’, ‘not helpful’ and ‘unapproachable’ to describe helpline volunteers. This is similar to the findings from a recent meta-synthesis of women’s experiences of breastfeeding support by Schmied and colleagues, who described breastfeeding support as occurring along a spectrum from an ‘authentic presence’ to ‘disconnected encounters’ [30]. They depict an effective supporter as having an ‘authentic presence’ (that is, taking a relationship-based approach, demonstrating empathy, taking time, and being affirming and responsive) and a ‘facilitative style’ (giving realistic and accurate information, practical help and encouragement) [30], qualities that were encountered by the vast majority of callers in our study. Our findings also reflect those from other qualitative studies of breastfeeding peer support which show that women value the shared understandings, emotional warmth, and time offered by mother-to-mother support [10,12-18]. The insights that peer support can impact upon breastfeeding continuation resonates with previous studies [18]. Furthermore, the findings that peer support provided callers with emotional benefits beyond breastfeeding support, such as reassurance and empowerment are similar to the findings from other studies of breastfeeding peer support [16,18]. This is also comparable to another recent evaluation of a breastfeeding helpline that identified ‘reassurance’, and increasing knowledge were among the key benefits reported by users [26].

The key barriers or difficulties experienced by callers in relation to their access and experience of helpline support concerned callers having to wait or call repeatedly before support could be accessed, and a lack of support during anti-social hours. As the majority of callers who use the helplines are first-time parents, it therefore appears essential that easily available help and support is provided, reinforcing the finding of Hoddinott et al. in terms of how women need to be able to access breastfeeding support at pivotal moments [31]. This finding suggests a need for more volunteers to operate the helpline and for an extended service to be offered; either earlier, later or on a 24-hour basis, with possible remuneration packages provided. Other key areas of concern related to occasions where volunteers were unable to answer the callers’ questions or callers receiving what they considered to be judgmental attitudes. The need for non-judgmental attitudes and support in caring for new mothers is well reported in the literature [30,32]. On occasions when follow-up support is warranted, or required by callers, call-back services could be provided by the volunteers. Ongoing training and support should also be provided to increase the knowledge base of volunteers, and to ensure that disapproving attitudes towards substitute feeding methods are not conveyed. A further and not surprising limitation relates to the remote nature of this support method; with numerous callers indicating the need for face-to-face support in resolving their breastfeeding difficulties. A further recommendation could therefore be to incorporate Skype or other remote/visual methods of communication to provide such a service. Consideration of the demographics also suggests that these helplines serve a particular demographic. Further research into why other groups, such as younger mothers, or those from other ethnic groups do not access such support requires further investigation. Additional targeted promotional materials and advertising of the service may also encourage wider access.

The limitations of this paper are that only 45% of callers who contacted the helpline over the recruitment period agreed to take part in the evaluation. A number of these calls may have related to callers who were not contacting the helpline with direct experiences (e.g. a mother or a member of her personal network) and therefore outside of the scope of this evaluation. However, it may be that only callers with more positive experiences were recruited to take part. Further research that employs suitable methods to remove this sampling bias is therefore warranted, e.g. through recruitment adverts placed on the NBH, ABM or BfN websites.

## Conclusion

Breastfeeding helplines are a popular method for callers to receive support and information; with the introduction of a National Breastfeeding Helpline in the UK leading to a substantial increase in helpline use. Overall satisfaction with the helpline support was high with the vast majority of callers recalling positive experiences. Callers valued the opportunity for accessible, targeted, non-judgmental and convenient support. Whilst the telephone support did not necessarily influence women’s breastfeeding decisions, the support they received left them feeling reassured, confident and more determined to continue, and in the majority of occasions the help received resolved their particular concerns. We recommend the helpline service be extended to ensure support can be accessed when needed, along with ongoing training and support for volunteers. Further advertising and promotion of the service within wider demographic groups is warranted.

## Competing interests

There are no financial or non-financial competing interests (political, personal, religious, ideological, academic, intellectual, commercial or any other) to declare in relation to this manuscript.

## Authors’ contributions

GT was involved in the design, data collection, analysis, reporting of the data and was lead author on the manuscript. NC was involved in data collection, contributed to data analysis and helped draft the manuscript. Both authors read and approved the final manuscript.
